# Aberrant salience network functional connectivity in auditory verbal hallucinations: a first episode psychosis sample

**DOI:** 10.1038/s41398-018-0118-6

**Published:** 2018-03-27

**Authors:** Pavan Kumar Mallikarjun, Paris Alexandros Lalousis, Thomas Frederick Dunne, Kareen Heinze, Renate LEP Reniers, Matthew R. Broome, Baldeep Farmah, Femi Oyebode, Stephen J Wood, Rachel Upthegrove

**Affiliations:** 1 Institute for Mental Health, University of Birmingham, Birmingham, UK; 2 College of Medical and Dental Sciences, University of Birmingham, Birmingham, UK; 3 Forward Thinking Birmingham, Birmingham, UK; 4 University of Oxford, Oxford, UK; 5 Birmingham and Solihull Mental Health Foundation Trust, Birmingham, UK; 6 Orygen, The National Centre of Excellence in Youth Mental Health, Melbourne, Australia; 7 Centre for Youth Mental Health, University of Melbourne, Melbourne, Australia

## Abstract

Auditory verbal hallucinations (AVH) often lead to distress and functional disability, and are frequently associated with psychotic illness. Previously both state and trait magnetic resonance imaging (MRI) studies of AVH have identified activity in brain regions involving auditory processing, language, memory and areas of default mode network (DMN) and salience network (SN). Current evidence is clouded by research mainly in participants on long-term medication, with chronic illness and by choice of seed regions made ‘a priori’. Thus, the aim of this study was to elucidate the intrinsic functional connectivity in patients presenting with first episode psychosis (FEP). Resting state functional MRI data were available from 18 FEP patients, 9 of whom also experienced AVH of sufficient duration in the scanner and had symptom capture functional MRI (sc fMRI), together with 18 healthy controls. Symptom capture results were used to accurately identify specific brain regions active during AVH; including the superior temporal cortex, insula, precuneus, posterior cingulate and parahippocampal complex. Using these as seed regions, patients with FEP and AVH showed increased resting sb-FC between parts of the SN and the DMN and between the SN and the cerebellum, but reduced sb-FC between the claustrum and the insula, compared to healthy controls.It is possible that aberrant activity within the DMN and SN complex may be directly linked to impaired salience appraisal of internal activity and AVH generation. Furthermore, decreased intrinsic functional connectivity between the claustrum and the insula may lead to compensatory over activity in parts of the auditory network including areas involved in DMN, auditory processing, language and memory, potentially related to the complex and individual content of AVH when they occur.

## Introduction

Auditory verbal hallucinations (AVH) are commonly linked to schizophrenia^1^ and occur in over 75% of the patients with First Episode Psychosis (FEP)^2,3^. Current biological models of AVH can be grouped into those based on abnormal brain activation, source-monitoring deficits, or errors in prediction, ^4–6^. The abnormal activation theory proposes that AVH arise from spontaneous activation in the primary auditory, superior temporal cortex and related memory areas^7–10^. According to source monitoring accounts, AVH results from failure to accurately monitor one’s own internal speech, which is then attributed to an external source^11^. Prediction error models are based on the Bayesian framework that proposes positive symptoms of psychosis including hallucinations arise due to a mismatch between prior expectations and incoming sensory information, leading to a prediction error and voice perception^6,12–14^. These models are not mutually exclusive and some commonality exists, for example prediction error can be implicated in source monitoring deficits. Study of brain activity during hallucination itself (symptom capture) allows direct evidence of regions active during hallucinations, and studies using this method show these regions include areas important for auditory perception (primary auditory cortex, and middle and superior temporal cortex), language (inferior frontal gyrus), and memory (hippocampus and parahippocampus)^13,14^. However, there remains a lack of detailed modelling that explains the spontaneous, complex and transient nature of AVH, and how the aberrant activity in these brain regions is triggered in the absence of an external stimuli^15^. Thus the key question of how the brain generates AVH remains unanswered.

Recently, theories of abnormal integration have been proposed to explain symptoms of schizophrenia, including delusions and hallucinations, with a central abnormality being aberrant activity in intrinsic brain networks such as the default mode network (DMN)^16,17^ or the salience network (SN)^18,19^. Northoff proposed that AVH may result from abnormally elevated resting state activity in auditory cortex or from the default-mode network, with a ‘neural confusion’ between auditory resting state stimulus-induced activity^20^, while Jardri et al. proposed that hallucinations occur during spontaneous DMN withdrawal^17^. Disordered connectivity has also been implicated in schizophrenia, with mechanism of aberrant SN co-ordination of DMN and other brain regions ^18,19^. Thus, there is growing evidence of aberrant DMN and SN activity in schizophrenia, and a failure of integration or altered connectivity within brain networks that may explain positive symptoms including delusions and hallucinations. However, additional evidence is needed to elucidate the relationship of this activity to hallucinations in particular. Previous investigations of AVH, predominantly in patients with schizophrenia and long standing illness, assessing functional connectivity (FC) have used a seed-based approach (sb-FC) based on the three hypotheses outlined above^5^, with seed placement in brain areas responsible for auditory processing^21–23^, language^22–25^, and memory^24^; the striatum^26^, and in areas of DMN^22,27,28^. These have generated some conflicting results^29^, possibly because of the varying seed placement and failure to control confounding factors such as length of illness and psychotropic medication^5^.

The aim of the current study was to address these confounding factors by investigating the intrinsic FC in FEP patients with AVH. We further aimed to improve selection of seed regions by identifying brain areas activated during AVH itself in the same sample. Based on previous literature in participants with chronic AVH outlined above, we hypothesised that symptom capture in FEP patients would identify areas active in AVH including but not limited to primary auditory cortex, inferior frontal gyrus and the insula and that patients would show aberrant resting state FC between areas of the DMN and SN and these areas.

## Materials and methods

### Participants

Eighteen individuals diagnosed with FEP were recruited from the Early Intervention Service and Youth Service, Birmingham and Solihull Mental Health NHS Foundation Trust within 12 months of initial diagnosis and treatment onset. FEP was defined as the first presentation of DSM-IV^30^ schizophrenia, schizophreniform disorder, schizoaffective disorder or psychotic disorder not otherwise specified, confirmed using M.I.N.I. International Neuropsychiatric Interview (M.I.N.I. 6.0)^31^. All had at least one episode of AVH every other day.

Age, gender and handedness matched healthy controls (HC) (*n* = 18) were recruited from the local community via advertisements. HC had no personal history of mental illness or current mental health problems, and no first-degree relative with a history of psychotic disorder as defined by self-report.

Exclusion criteria for both groups included a history of any neurological disorder, seizures, or significant head injury, any contraindications for MRI and any significant risk history including suicide risk and violence (as assessed by the clinical team).

Ethical approval was obtained from the NHS HRA (reference 13/WM/0277) Ethics Committee. All participants provided written informed consent to participate in the study.

### Procedure, MRI acquisition and preprocessing

Information on demographic and clinical variables including age, gender, marital status, duration of treated and untreated psychosis, and current medication was recorded for patients. AVH were comprehensively assessed for both frequency and quality using the Beliefs about Voices Questionnaire—Revised^32^ (BAVQ-R), Topography of Voices Rating Scale^33^ (TVRS), Voice Power Differential Scale^34^ (VPD), and Auditory Hallucinations Subscale of Psychotic Symptoms Rating Scale^35^ (PSYRATS).

### MRI acquisition

Patients underwent neuroimaging including fMRI-symptom capture, resting state and structural MRI at the Birmingham University Imaging Centre using a 3 T Philips Achieva MRI scanner, whilst the HC completed the resting state and structural scan. Patients completed a 10-min fMRI symptom capture run wherein 200 volumes of BOLD-fMRI were acquired (TE = 30 ms, whole-brain coverage, TR = 3 s, voxel size 3 mm × 3 mm × 2 mm, field of view 192 × 192 × 100, flip angle 90°, matrix 64 × 64). Throughout this scan, participants had their right thumb resting on a button; and they were instructed to press and hold this for the duration of any AVHs experienced.

Participants completed a 10-min resting state scan when they were in the scanner in a state of wakeful rest with their eyes closed. Two hundred volumes of BOLD-fMRI were acquired (TE = 30 ms, whole-brain coverage, TR = 3 s, voxel size 3 mm × 3 mm × 2 mm, field of view 192 × 192 × 100, flip angle 90°, matrix 64 × 64). T1-weighted images (TR = 8.4 ms, TE = 3.8 ms, flip angle = 8°, FOV = 288 × 232 × 175 mm, voxel size 1mm^3^) were acquired for each participant for image registration. Participants wore headphones and earplugs throughout both scan runs to attenuate the noise of the scanner.

### Preprocessing

Anatomical and functional MRI scans were preprocessed with statistical parametric mapping software (SPM 8, Friston, The Welcome Department of Cognitive Neurology, London, UK; http://www.fil.ion.ucl.ac.uk/spm) and the Data Processing Assistant for Resting-State fMRI Advanced Edition (DPARSFA) V3.1 (http://rfmri.org/DPARSF)^36^.

Preprocessing of symptom capture data: Preprocessing of the acquired images included slice time correction, realignment, co-registration with the anatomical image, spatial normalisation using a Montreal Neurological Institute template scan, and smoothing using 8-mm full-width-at-half-maximum 3D Gaussian kernel. If excessive movement was noted of realignment, defined as a translation of > 1 voxel (3 mm × 3 mm × 2 mm) or rotation of >2 degrees, artefact repair was performed to correct for it using the ArtRepair toolbox^37^.

Preprocessing of resting state scans: The first ten volumes from each scan were excluded as dummy scans to allow stability of longitudinal magnetisation. The images were then reoriented and corrected for slice timing differences, with the middle slice being used as a reference slice. Both the T1 and the functional images were reoriented in order to improve co-registration accuracy. Brain extraction was then performed on the T1 images in order to remove the skull before co-registration to the functional images and to improve the co-registration algorithm. The T1 images were then coregistered to the functional images and segmented into grey matter, white matter (WM), and cerebrospinal fluid (CSF) partitions. The Friston 24-parameter model^38^ was then used to obtain six head motion parameters, six head motion parameters one time-point before, and the 12 corresponding squared items and input them into our model as nuisance regressors. Any time-points that had a framewise displacement^39^ of more than 0.5 as well as one time-point before them and two time-points after them were regressed out. The WM and the CSF masks that were created during segmentation were then combined and their time series was calculated. Then, their first five principal components were calculated and entered as nuisance regressors in the model (CompCor)^40^. Diffeomorphic Anatomical Registration using Exponentiated Algebra (DARTEL)^41^ was used to create a group specific template to which all of the images were normalised to. An affine transformation of the images to the Montreal Neurological Institute (MNI) stereotactic space using the parameters estimated in DARTEL was performed. Finally, the images were smoothed with a Gaussian kernel of 8 mm full-width at half maximum.

### Data analysis

Statistical analyses of demographic and clinical data were performed using SPSS (SPSS version 24, Chicago, IL, USA). After testing for the normality of the distributions, continuous variables were compared using *t*-tests and categorical variables were compared using chi-square tests. Significance was set at *p* < 0.05.

#### Symptom capture analysis

General linear model analysis was used on the pre-processed images. For each subject, one contrast vector (AVH minus rest) was specified to evaluate the effect of AVH (button pressed) relative to the baseline (button not pressed) with the six movement parameters (three translation and three rotation) from pre-processing entered as nuisance regressors. This produced a contrast image for each subject that was then entered into a second level, random-effects analysis, using a one-sample *t*-test. A group comparison of activation during AVH and at rest was then conducted. A cluster was considered as significant at *p* < 0.01 (uncorrected) and an extent criterion of *k* = 60 to reduce the likelihood of identifying false positive clusters. The uncorrected threshold was chosen to reduce the likelihood of type 2 error.

#### Seed-based FC analysis

The brain regions that were identified in the symptom capture study were included as regions of interest (ROI) for the FC analyses. ROI were created using spherical peak coordinates with a radius of 4 mm. Four millimetre radius was chosen over 6 mm, more frequently used, in the aim of adding specificity to regions chosen. Pearson’s correlations coefficients between the time series of each seed region with voxels of the rest of the brain were then calculated for each ROI. A Fisher transformation was then used to convert these voxel-wise Pearson correlation coefficients into whole-brain *z*-values for each participant to conduct second-level analyses in SPM8.

The FC maps from individual participants were separately analysed using one-sample *t-*test for the entire sample with cluster level significance level set at *p* < 0.05 (family-wise error (FWE) corrected). The results from the one sample *t-*test were used to derive search volume masks for the FC to constrain subsequent between group analysis. The masks represented regions with significant instantaneous positive correlation or anticorrelation with the seed region. Between group analyses were conducted using an unpaired *t*-test with significance set at *p* < 0.05 (FWE corrected). All group level analyses were carried out using SPM8 software and the toolboxes MarsBar (http://marsbar.sourceforge.net) and xjview (http://www.alivelearn.net/xjview8).

In the patient group, bivariate correlations were used to examine the influence of current antipsychotic medications (chlorpromazine equivalent), and duration of illness on the mean coefficients within the clusters that emerged as significant from the two-sample *t*-tests. The scores on severity of hallucinations (TVRS, VPD, PSYRATS) were correlated with the mean coefficients within the clusters that were significantly different between patients and controls. The significance level was set at *p* < 0.05 (FWE corrected across all comparisons).

## Results

### Demographic and clinical data

Clinical data analyses are summarised in Table 1. Nine individuals with FEP were diagnosed with schizophrenia, four with psychotic disorder not otherwise specified, one with schizoaffective disorder, four with schizophreniform disorder. Median dose of antipsychotic medication currently taken in chlorpromazine equivalents was 200 mg (range 0–400 mg). Two patients were not on any antipsychotic medication. The mean duration of treated psychosis was 103 days (median 99, range 0–360) and of untreated psychosis was 226.5 days (range 6–1095 days).

**Table 1:** Demographic and clinical characteristics of the sample

	FEP patients with persistent AVH (*n* = 18)	Healthy controls (*n* = 18)	*p* value
Age (mean ± s.d.)	27.14 ± 5.66	26.94 ± 4.31	0.91
Gender (Male/female)	15/3	15/3	0.73
Marital status (single/married)	18/0	16/2	0/492
CPZ equivalents (mean ± s.d.)	217.85 ± 139.51		
Duration of untreated psychosis (days) (mean ± s.d.)	323.92 ± 311.23		
BAVQ-*R* scores			
Malevolence (mean ± s.d.)	11.07 ± 4.54		
Benevolence (mean ± s.d.)	4.00 ± 3.28		
Omnipotence (mean ± s.d.)	11.57 ± 4.32		
Resistance (mean ± s.d.)	19.36 ± 3.67		
Engagement (mean ± s.d.)	5.21 ± 3.98		
TVRS scores (mean ± s.d.)	17.71 ± 3.47		
VPDS scores (mean ± s.d.)	22.93 ± 5.44		
PSYRATS (auditory hallucination subscale)			
Total score (mean ± s.d.)	26.86 ± 4.95		
Emotional domain (mean ± s.d.)	11.29 ± 3.42		
Cognitive domain (mean ± s.d.)	8.64 ± 1.82		
Physical domain (mean ± s.d.)	9.79 ± 1.92		

*FEP* first episode psychosis, *AVH* auditory verbal hallucinations, *CPZ* chlorpromazine, *BAVQ-R* belief about voices questionnaire—revised, *TVRS* topography of Voices Rating Scale, *VPDS* Voice Power Differential Scale, *PSYRATS *Psychotic Symptom Rating Scales

### Symptom capture analysis

The results of the symptom capture analysis are presented in Table 2. Twelve of the 18 patients with AVH reported hearing voices during the symptom capture scan. The number of AVHs experienced by each participant during the scan time ranged from 1-17 with a mean number of 6.22 (SD 6.00) and median of 3 (IQR 10.00). Individual AVH duration ranged from 4.17–154.93 s with a median of 9.06 s (IQR 11.65). Individual participants’ cumulative duration of AVHs ranged from 4.17–240.83 s with a median of 127.83 s (IQR 121.98). Nine of the 12 patients’ images were used for analysis as 3 patients only had AVH which lasted less than 4 s, thus as the delay in BOLD response is around 8 s, their AVH did not outlast the BOLD response.

**Table 2:** Coordinates of brain regions activated during auditory verbal hallucinations (*n* = 9)

Region of interest (BA)	MNI Coordinates (*xyz*)	Cluster size,	*p* value (uncorr)
Cluster 1 (left)			
Lingual gyrus (18)	-24 -58 -4	*k* = 121	*p* < 0.001**
Lingual gyrus (19)	-18 -46 -8		
Parahippocampal gyrus (19)	-33 -55 -4		
Cluster 2 (left)	-48 2 -6	*k* = 61	*p* = 0.013
Insula (22)	-54 11 -8		
Superior temporal gyrus (22)	-54 -7 -10		
Superior temporal gyrus (22)			
Cluster 3 (right)			
Superior temporal gyrus (22)	51 -7 -2	*k* = 141	*p* < 0.001**
Insula	42 -19 -6		
Superior temporal gyrus (22)	48 -16 -14		
Cluster 4 (left)			
Claustrum	-33 -7 -10	*k* = 65	*p* = 0.010
Insula (13)	-45 -13 -4		
Claustrum	-39 -1 -8		
Cluster 5 (right)			
Precuneus (31)	12 -64 22	*k* = 61	*p* = 0.013
Precuneus (31)	24 -70 20		
Posterior cingulate cortex (31)	21 -61 14		

*BA* Broadman area

**significant at FDR < 0.05

The brain regions activated during AVH compared to rest included bilateral auditory processing areas (superior temporal cortex), bilateral insula, posterior regions of DMN (precuneus and posterior cingulate cortex) and lingual/ parahippocampal complex (Table 2).

### Seed-based FC analysis

The areas of activations from the symptom capture study formed the 15 ROI seeds (see Table 2) for the FC analysis. Four subjects had excessive head motion (translation >2.0 mm and rotation >2°) at pre-processing and were removed from the sb-FC analysis.

**Fig. 1:**
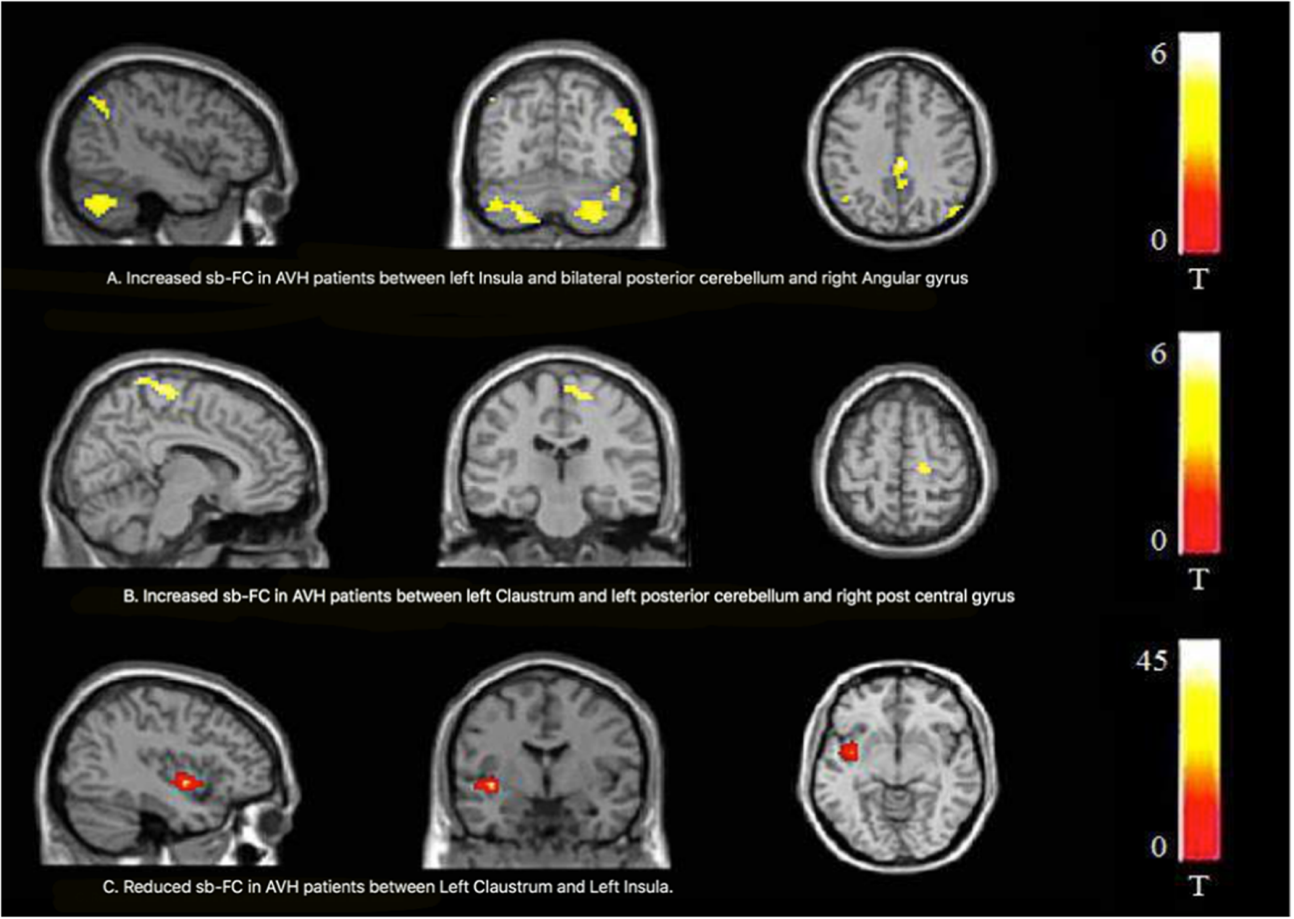
Whole-brain voxel-based comparisons of the Insula/Claustrum functional connectivity in schizophrenia patients with auditory verbal hallucinations (AVH, *n* = 14) and matched healthy volunteers (HC, *n* = 18). Contrast maps are overlaid on a structural MRI brain (*n* = 32; *P*_corr_ 0.05). Increased sb-FC was detected in patients between left Insula and bilateral posterior cerebellum and angular gyrus (**a**). Increased sb-FC was detected in patients between left Claustrum and left posterior cerebellum and right post central gyrus (**b**). A decreased sb-FC was measured in patients between left claustrum and left insula (**c**). Colour bars show a scale of *t* values

There was a statistically significant difference between patients with AVH and HC in FC on 2 of the 15 seed regions. The left insula and left claustrum seeds showed significant differences between patients with AVH and HC (Table 3 and Fig. 1).

**Table 3:** Differential functional connectivity (FC) of Insula and Claustrum between patients with AVH (*n* = 14) and Healthy controls (*n* = 18)

Seed region		Brain region (BA)	MNI coordinates			Cluster size (mm^3^)	Tmax	*p* corr value^a^
			*X*	*Y*	*Z*			
L Insula	AVH > HC	R Posterior cerebellum	30	−75	−45	406	5.94	<0.001
		R Posterior cerebellum	42	−78	−33			
		R Angular gyrus (39)	57	−66	30	125	5.06	0.026
		R Angular gyrus (39)	60	−57	30			
		L Posterior cerebellum	−21	−75	−48	210	4.76	0.003
L Claustrum	AVH > HC	L Posterior cerebellum	−15	−84	−42	121	4.94	0.012
		L Posterior cerebellum	−39	−69	−39			
		L Posterior cerebellum	−48	−63	−48			
		R Postcentral gyrus (5)	6	−51	75	134	4.72	0.007
L Claustrum	HC > AVH	L Insula (13)	−39	−3	−6	101	4.42	0.024

*BA* Broadman area, *L* left, *R* right

^a^ Family-wise error (FWE) corrected

Patients showed increased FC between left insula and bilateral cerebellum, and angular gyrus; and increased FC between left claustrum and left cerebellum and postcentral gyrus. There was reduced FC in FEP patients with AVH between left claustrum and left insula compared to HC. The FC between left insula and left claustrum seeds for patients and HC is shown separately in supplementary information (Tables 1–4 in supplementary information).

There were no significant correlations between DUP, dose of antipsychotic medications, and severity of hallucinations and the mean coefficients of clusters that were significantly different between FEP patients and HC.

## Discussion

This is the first study of FC in FEP patients with frequent AVH and matched controls, using seed regions obtained during symptom capture in the same group. Thus, we report results with reduced confounding factors of chronicity of illness, repeated episodes, long duration of treatment and model based selection of seed regions. The results from symptom capture showed that the bilateral superior temporal cortex, precuneus/posterior cingulate cortex, bilateral insula and parahippocampal regions were active during AVH. Using these areas as seed regions, we found that, compared to HC, FEP patients with frequent AVH had increased FC between left insula and bilateral cerebellum and angular gyrus; and increased FC between left claustrum and left cerebellum and postcentral gyrus. We also found reduced FC between left claustrum and left insula in FEP with AVH patients compared to HC. Contrary to our hypotheses, we found no significant sb-FC results from seeds placed in auditory processing areas [bilateral superior temporal gyrus (STG)], areas involved in memory (parahippocampal gyrus and lingual gyrus), or posterior DMN (precuneus and posterior cingulate cortex).

Thus, our main results include increased FC between left insula (a key node of SN^18,42^) and the angular gyrus, a region which has been consistently identified as a key parietal node of the DMN^43–45^. It is possible the pathology of AVH is primarily located in these two regions; the insula and angular gyrus. Our results of both the left insula seed shows connectivity with right insula and anterior cingulate cortex (key regions of SN). Together with literature from patients with chronic AVH, the suggestion may be that resting state dysconnectivity within the DMN and SN are implicated in the generation of AVH, which during the experience itself will further involve temporal and auditory networks^28^. Reduction in left insula volume has been observed in patients with schizophrenia and prominent hallucinations^46^ and volumes of the left insula and right STG have been negatively correlated with severity of AVH in a meta-analysis of structural studies^47,48^. Although with a small sample and cross sectional study it is speculative to propose definitive models, our results suggest that AVH possibly begin with dysconnectivity between the DMN and SN. AVH are then further developed by the incorporation of other regions resulting in a ‘heard’ experience or one associated with memory, as our symptom capture results confirm activation in the superior temporal cortex, and parahippocampal areas during AVH itself.

We also found increased FC in FEP with AVH between the left insula and the bilateral posterior cerebellum; and between the left claustrum and the left posterior cerebellum. Additionally, we found reduced sb-FC between the left claustrum and the left insula in FEP with AVH in comparison to HC, which has not been reported previously. The claustrum has been reported to be activated specifically during AVH^49^. The role of the claustrum includes sensory binding^50^, modulation of selective attention^51^, synchrony detection, and modulation^52^ and switching between intrinsic networks^53^. Reduced FC between the claustrum and the insula may indicate a reduced input of multi-sensory integrated information from claustrum to insula, which may impact on proximal salience appraisal.

Contrary to our findings, Clos et al.^22^ previously found reduced connectivity in several brain regions in patients with AVH including between right cerebellum and left thalamus. However this may be the result of different methodology (which involved replicating voice experience with playing recorded speech-like sounds) and choice of seed placement. Our findings suggest a greater role for the insula in AVH which, along with anterior cingulate cortex and ventral striatum, has been shown to be an important brain region involved in prediction error coding^54,55^. Aberrant predictive coding error could be a mechanism by which the SN dysfunction leads to AVH. Timing, sensory prediction and learning are involved in perceptual processes, are key functions of the cerebellum^56,57^ which may have aberrant FC in patients with AVH. The cerebellum is part of several intrinsic brain networks including the SN and the DMN^58^, and our results add to the evidence for dysconnectivity involving the cerebellum in the generation of AVH.

We tentatively propose a unitary model of AVH, building on previous literature and our preliminary findings, with aberrant connectivity between the SN and DMN essential in AVH generation. The SN is necessary to initiate and modify sensory information and action^59^ and mediates the switching between the DMN and the task positive network^60^. In this model, insula dysfunction is essential in the generation of AVH via evaluation of stimuli and attribution of salience to them. Aberrant activity within the claustrum–insula complex may be involved in impaired proximal salience appraisal leading to AVH, with compensatory activity in other areas, including the auditory network and areas involved in auditory processing, language and memory, and areas involved network dysconnectivity in predictive coding (cerebellum). This model could incorporate the abnormal integration, source monitoring and prediction error theories. Future studies would be needed to elucidate the temporal relationship this model suggests.

The results of this present study and any model suggested should be interpreted with caution due to clear limitations. These include primarily the small sample size, particularly of participants included in the symptom capture analysis, however this is comparable with previous fMRI symptom capture studies in chronic schizophrenia, which number 1–18, and reflect the challenge of capturing AVH in real time ^13^. We have been cautious in interpretation of our results therein. In addition, not all of the participants experienced hallucinations during the symptom capture part of the study and thus it is possible that these participants had different brain regions active during their AVH that were not captured in our seed placement for sb-FC. Patients included in the symptom capture analysis had a cumulative median duration of AVH of 127 s (out of 600 s of scan time). This along with our less stringent use of uncorrected cluster level significance of *p* < 0.01 may suggest that this study was underpowered with a potential for type 2 error. Notwithstanding, the results of the symptom capture have identified similar areas as the meta-analysis by Jardri et al.^13^ which provides some validity to the results from the symptom capture analysis and suggest the changes seen in chronic schizophrenia are also present early in the course of illness. Future studies should include a clinical control group of patients with psychosis who are not experiencing AVH to definitively establish the specificity of results to AVH rather than other group characteristics. Using seed regions generated during symptom capture provide validity to a regions’ involvement in AVH, however with 15 seeds being used for sb-FC analysis, there is a possibility of type 1 error. To give some address to this we have corrected for multiple comparisons using family-wise error correction within each analysis. Finally, although we have found no correlation with our FC results and medication, given the majority of participants were medicated cannot rule out effects entirely.

In summary, in FEP patients with frequent AVH and reduced confounds of long duration of treatment, repeated episodes or length of illness, there was some evidence of aberrant FC between the SN and the DMN; the SN and the cerebellum; and the SN and the claustrum. This may provide some basis of a unified mechanism for the generation of AVH, with the claustrum/ insula as a novel target area of interest. Future research should focus on the strength and directionality of connectivity between these regions and the temporal relationship between this and auditory processing, language and memory areas to explore the specificity of this aberrant FC to the detailed and varied experience of voice hearing.

## Electronic supplementary material

Supplementary figure 1, 2 and tables 1-4(DOCX 5945 kb)
