# Supplementary material for: Aberrant salience network functional connectivity in auditory verbal hallucinations: a first episode psychosis sample
Source: Transl Psychiatry. 2018 Mar 27;8:69. doi: 10.1038/s41398-018-0118-6 (PMC5913255; doi:10.1038/s41398-018-0118-6)
Supplement: Supplementary file 1 — Supplementary figure 1, 2 and tables 1-4(DOCX 5945 kb) [file 41398_2018_118_MOESM1_ESM.docx]

Supplementary figure and tables:

**Figure 1:** Whole brain voxel based comparisons of the left Insula connectivity in patients with AVH and matched healthy controls. Colour bars show a scale of T values.

**Figure 2:** Whole brain voxel based comparisons of the left Claustrum connectivity in patients with AVH and matched healthy controls. Colour bars show a scale of T values.

**Table 1:** Whole brain sb-FC for Insula seed in patients with AVH (n=14)

| *sb-FC of the Left Insula Seed (Patients)* | | | |
| --- | --- | --- | --- |
| Brain Region (Broadman areas) | MNI Coordinates | P Value, Peak Intensity, Cluster Size |  |
| Right Cerebellum | 15 -87 -45 | p(FWE)=0.005, T=5.16, k=189 |  |
| Left Cerebellum | -33 -66 -39 | p(FWE)=0.001, T=5.42, k=268 |  |
| L Superior Temporal Gyrus (BA22) | -48 0 -6 | p(FWE)<0.001, T=42.28, k=2911 |  |
| R Insula (BA13) | 36 -9 -18 | p(FWE)<0.001, T=6.45, k=2143 |  |
| Middle Frontal Gyrus (BA 10) | -36 48 33 | p(FWE)=0.001, T=5.24, k=257 |  |
| Medial Frontal Gyrus (BA 6) | 6 6 51 | p(FWE)<0.001, T=5.97, k=805 |  |

**Table 2:** Whole brain sb-FC for Insula seed in healthy volunteers (n=18)

| Brain Region (Broadman areas) | MNI Coordinates | P Value, Peak Intensity, Cluster Size |
| --- | --- | --- |
| L Superior Temporal Gyrus (BA22) | -48 0 -6 | p(FWE)<0.001, T=57.01, k=2840 |
| R Superior Temporal Gyrus (BA 22) | 57 0 -6 | p(FWE)<0.001, T=7.70, k=2554 |
| R Medial Frontal Gyrus | 3 12 54 | p(FWE)<0.001, T=7.33, k=1065 |

**Table 3**: Whole brain sb-FC for Claustrum seed in patients with AVH (n=14)

| Brain Region (Broadman Area) | MNI Coordinates | P Value, Peak Intensity, Cluster Size |
| --- | --- | --- |
| L Insula (BA 13) | -39 -3 -9 | p(FWE)<0.001, T=30.49, k=2985 |
| R Superior Temporal Gyrus (38) | 51 3 -18 | p(FWE)<0.001 T=7.98, k=2747 |
| L Middle Frontal Gyrus (BA 10) | -33 45 15 | p(FWE)=0.001, T=5.80, k=198 |
| L Medial Frontal Gyrus (BA 6) | 3 9 45 | p(FWE)<0.001, T=10.80, k=1131 |
| L Precentral Gyrus (BA 6) | -36 -15 63 | p(FWE)=0.004, T=5.33, k=149 |

**Table 4**: Whole brain sb-FC for Claustrum seed in healthy volunteers (n=18)

| *sb-FC of the Left Claustrum Seed (Healthy Controls)* | | | |
| --- | --- | --- | --- |
| Brain Region (Broadman Areas) | MNI Coordinates | P Value, Peak Intensity, Cluster Size |  |
| L Insula (BA13) | -39 -3 -6 | p(FWE)<0.001, T=90.03, k=3329 |  |
| R Insula (BA 13) | 39 -6 6 | p(FWE)<0.001 T=7.85, k=2386 |  |
| Medial Frontal Gyrus (BA 6) | 3 9 45 | p(FWE)<0.001, T=7.81, k=1093 |  |
